# Julie Makani: leveraging innovation to tackle sickle cell disease

**DOI:** 10.2471/BLT.24.031024

**Published:** 2024-10-01

## Abstract

Julie Makani talks to Gary Humphreys about the need for guidance and policy to reflect developments in treatment of sickle cell disease.

*Q: Until recently, advances in research and treatment for sickle cell disease were not reflected in guidance adapted for the African region where an estimated 80% of worldwide cases occur. What do you think accounts for this situation?*

A: First, you are right to point out that the guidance situation did recently improve. In June 2024, the WHO (World Health Organization) African Regional Office published a sickle cell disease technical package that provides a comprehensive and integrated approach to managing the disease. The guidance includes recommendations for the use of hydroxyurea and rapid diagnostic tests, two key interventions that have been demonstrated to be of enormous value. However, it is also true to say that those interventions have been available for some time. Indeed, the therapeutic value of hydroxyurea in the treatment of sickle cell disease was first demonstrated in the trials conducted by Dr Michael B Vichinsky in the late 80s and 90s, even if regulatory approval came later. As to why it has taken so long for hydroxyurea treatment to feature in guidance, I believe it reflects – in part at least – the general neglect of this disease by the research community. Without research, there is no evidence base or, for that matter, regulatory approval, and without that base, no guidance. Despite sickle cell disease imposing a tremendous burden of disease, with an estimated eight million people affected worldwide, it has been largely neglected. Even in Africa, where it is estimated that around 240 000 children are born with sickle cell disease every year, up to 80% of whom will die from severe infections or acute chest syndrome before age five, tackling the disease is still not prioritized in many countries. From researchers’ point of view, the sad reality is that there is little incentive to go into the field and this has been the case for decades. When I completed my clinical training in the late 90s and expressed a desire to work on sickle cell disease, people said: “Why would you enter a field with no funding, no opportunities and no future?” Fortunately, Muhimbili University and Oxford University were both very supportive and that support allowed me to pursue my work.

Another issue is the lack of connection between research and policy. Again, I have personal experience of this mismatch. I was always told that building a scientific career was about securing grants and publishing papers, not improving health care or engaging with policy-makers. And when I questioned these assumptions, saying I wanted to impact health care directly, some people questioned whether I was truly committed to being a scientist.

“Robust, evidence-based normative guidance is crucial.”

My desire to train doctors and set up postgraduate training in haematology was met with similar scepticism, as were my efforts to engage with national policy-makers, including the Tanzanian Ministry of Health. At the institutional level, fragmentation and silo-thinking is a major obstacle. If you want to discuss newborn screening for sickle cell disease, you’re directed to child health experts. If you want to talk about lab diagnosis, you're told to speak to diagnostic specialists. There’s little communication or coordination between these groups and that also impacts policy formation.

*Q: You have been able to focus considerable attention on sickle cell disease as a scientist, clinician, teacher and advocate. Have things not improved for would-be researchers?*

A: Not that you would notice. For example, I’ve been a visiting professor here at Imperial College London for a year, and I’m afraid to say I’ve observed a complete absence of opportunities for establishing a career in academia or research focused on sickle cell disease. One of the knock-on effects of this is a lack of experts in the field. This is problematic for all kinds of reasons, notably in terms of generating new ideas and fresh perspectives. Conversations can become stale, ideas can stagnate, including ideas among clinicians regarding treatment. The use, or rather underuse, of hydroxyurea in the treatment of sickle cell disease is a good example.

*Q: How is hydroxyurea used in the treatment of sickle cell disease?*

A: Sickle cell disease is a hereditary blood disorder caused by a mutation in the gene responsible for producing haemoglobin, the protein that carries oxygen around the body. As a result of this mutation, instead of being round and flexible, the red blood cells become sticky and sickle-shaped, which can lead to obstructions in small blood vessels, entraining various health problems. Hydroxyurea works by inhibiting DNA synthesis, which affects rapidly dividing cells, including cancer cells and bone marrow cells that produce blood cells. In sickle cell disease, the main therapeutic effect of hydroxyurea is increasing fetal haemoglobin which helps reduce the sickling of red blood cells. However, it also improves blood flow, reduces inflammation and decreases the frequency of painful crises. It can be administered to patients in the form of capsules or tablets and is relatively affordable at around 20 United States dollars (US$) to US$ 60 per month of treatment – although efforts to reduce costs and increase access continue. As I said at the outset, the therapeutic value of hydroxyurea in the treatment of sickle cell disease has long been understood and when we started our haematology clinic at Muhimbili, we were using it to treat patients with chronic myeloid leukemia. However, we found that health-care providers didn’t want to use it for sickle cell disease. We would hear things like, “Oh, this is a chemotherapy drug,” or “What about the side effects?” These and other concerns contributed to widespread under-utilization of what is in fact a safe and effective drug. Educating health-care professionals on the benefits and management of hydroxyurea through robust, evidence-based normative guidance is crucial for overcoming these obstacles. Hopefully, the new WHO guidance will make a big difference.

*Q: What other obstacles stand in the way of effective treatment of sickle cell disease?*

A: The absence of quality essential services in many health systems. Addressing sickle cell disease requires a comprehensive approach rooted in primary health-care delivery that includes widespread screening and preventive genetic counselling. Families with a history of sickle cell disease or sickle cell trait (which occurs when a person inherits one normal haemoglobin gene and one sickle cell gene**)** have a risk of passing the gene to future generations. Genetic counselling can help people understand the risks and make informed decisions. Implementation of newborn screening is also vital and, where appropriate, blood transfusions, bone marrow transplants and any other necessary treatments. But I want to underline the fact that addressing this disease goes beyond the delivery of health-care interventions; it’s about caring for people in the broader sense. When we first set up the clinic in 2003, we estimated that we would enrol 400 patients in the first year, but within a short period we had enrolled 5000, despite the fact we had very little to offer in terms of interventions. People came to us because we offered a space where patients felt heard and understood – not dismissed or stigmatized.

*Q: What drives stigmatization in relation to this disease?*

A: The short answer is lack of understanding, including misconceptions about chronic pain and opioid use. In some settings, racial discrimination is a factor. Making progress on the stigmatization front boils down to supporting education and outreach. Again, effective guidance can play a part in informing health-care and education policies, and promoting greater community and indeed societal awareness.

*Q: There have been several breakthroughs regarding the treatment and diagnosis of sickle cell disease in recent years. How can the drafters of normative guidance support their uptake and use?*

A: By developing guidance adapted for local use and ensuring its uptake in national policy. It’s important to realize however that there’s a limit to what guidance can achieve. In some cases, new treatments are, for the time being at least, unaffordable. In vivo gene therapy is a prime example. The United States Food and Drug Administration approved two milestone treatments in December 2023, but they cost millions of dollars per treatment. Providing in vivo gene therapy in sub-Saharan Africa will require not just a lowering of cost but also overcoming infrastructure, regulatory, ethical and logistical barriers. However, I am hopeful that given necessary investment of intellectual and material capital we can realistically aim for in vivo gene therapy as a goal in Africa.

Other examples include exchange transfusion, where sickled red blood cells are replaced with healthy donor blood cells, and bone marrow transplants. Because these therapies are expensive, there is a tendency to overlook them in guidance and policy development, but opportunities are perhaps being missed. Just because treatments are expensive does not stop patients demanding them or suppliers making them available. For example, health facilities outside Africa are using social media to advertise transplants to cure sickle cell disease priced at US$ 50 000. Some patients are responding to this, and not just the wealthy. I know a mother whose daughter survived two near-fatal sickle cell disease-related acute chest syndrome episodes, who sold her property to fund the treatment. Out-of-pocket expenditure at this level clearly presents problems for households, but health systems are also impacted – for example when such procedures lead to complications which must then be addressed. And this is not just happening in the United Republic of Tanzania; we are seeing cases in Nigeria, Uganda and Zambia where patients leave to get transplants, then return and struggle to obtain post-transplant care because the health systems are not prepared. Some governments are starting to respond, including Nigeria, South Africa and the United Republic of Tanzania, all of which have established bone marrow transplant services now. Whether the provision of such services will be sustainable in the long run remains to be seen, and once again some evidence-based guidance, in this case on health system financing, would be helpful.

*Q: Where do you believe health authorities should be focusing their efforts for now?*

A: On making the best use of the tools we already have. The introduction of point-of-care tests has transformed our approach to diagnosis at the primary care level. The affordability of hydroxyurea is also improving, and will continue to do so as more countries add it to their essential medicines lists and implement pooled procurement strategies. My hope is that governments will take notice, and drive the policy development needed to ensure optimal implementation of the interventions available while investing in the health systems needed to deliver them.

